# Editorial: Focus on malignant pleural mesothelioma immunology and immunotherapy

**DOI:** 10.3389/fimmu.2023.1251384

**Published:** 2023-07-24

**Authors:** Chiara Porta, Didier Jean, Christophe Blanquart

**Affiliations:** ^1^ Department of Pharmaceutical Sciences, University of Eastern Piedmont, Novara, Italy; ^2^ Center for Translational Research on Autoimmune & Allergic Diseases (CAAD), University of Eastern Piedmont, Novara, Italy; ^3^ Centre de Recherche des Cordeliers, INSERM, Université Paris Cité, Sorbonne Université, Functional Genomics of Solid Tumors Laboratory, Paris, France; ^4^ Centre National de la Recherche Scientifique (CNRS), Paris, France; ^5^ Institut National de la Santé et de la Recherche Médicale (INSERM) U1232 Centre de Recherche en Cancérologie et Immunologie Nantes Angers (CRCINA), Nantes, France

**Keywords:** mesothelioma, immune checkpoint inhibitors, predictive biomarkers, tumor microenvironment, eosinophils, microbiota, osteopontin, oncolytic vaccinia virus

Malignant pleural mesothelioma (MPM) is a rare and aggressive thoracic cancer that derives from the mesothelial cells of the pleura and is causally associated with exposure to asbestos. Because of the poor specificity of the clinical symptoms, when it is diagnosed, malignant cells, which are extremely resistant to therapies, have already spread throughout the pleural layers, leading to a poor outcome. Although the recent approval of the combination immunotherapy with anti-CTLA-4 and anti-PD-1 represents a breakthrough for MPM, many patients are still refractory or relapsed after a few months of therapy. Therefore, there is an urgent need of reliable biomarkers to improve patient selection for immune checkpoint blockades (ICBs), as well as of new therapeutic strategies to boost anti-tumor immunity. Recent insights into these unmet medical needs are discussed in this Research Topic. Specifically, Perrino et al. review the clinical efficacy and the most promising predictive biomarkers of response to ICBs in MPM. In addition to widely recognized determinants - such as PD-L1 expression and tumor mutational burden (TMB) - and specific prognostic factors for MPM (i.e. histological subtype), they discuss new elements, which definitively warrant to be further investigated and prospectively validated. Particularly, it is worth noting that in spite of the low TMB, MPM cells usually show multiple chromosomal rearrangements, which can lead to the expression of neo-antigens, thereby predicting response to ICBs.

Besides histological classification in epithelioid, sarcomatoid and biphasic MPM, a growing number of studies have pointed out a striking molecular heterogeneity, which suggests the existence of a range of molecular phenotypes associated with different responsiveness to ICBs and outcome. Based on these premises, the studies of Yang et al. and Liu et al. generate in-silico classification systems for MPM, which could be exploitable to guide immunotherapy strategies, provided that those results will be validated in prospective studies and larger cohorts. Specifically, Yang et al. provide a machine learning-based 12-gene classifier to separate MPM in two immune-related subtypes. That one associated with a better response to ICBs is the immune activated subtype, which harbors an IFN-γ dominant immune phenotype, a consistent TCR and BCR diversity and the highest lymphocyte infiltration. Conversely, Liu et al. elaborate a classification system based on the expression of damage-associated molecular patterns (DAMPs). In this regard, the “inflammatory DAMPs subtype”, which is characterized by the enrichment of proinflammatory cytokine signaling, is also associated with better outcome.

Alongside the analysis of tumor microenvironment (TME) composition, liquid biopsy is emerging as an interesting research area for predictive biomarkers of response to ICBs. Eosinophils can support local anti-tumor response by producing cytotoxic molecules, but they can also secrete cytokines promoting suppressive macrophages, which are both the major component of the MPM microenvironment and limit for ICB success. Willems et al. demonstrate a correlation between a baseline absolute eosinophil count (AEC) of ≥220/µL and a worse outcome of MPM patients undergoing chemo- or immunotherapy. Thus, further prospective studies are warranted to validate blood AEC as a potential predictive biomarker for both therapies.

Microbiome is a key environmental determinant of ICB efficacy for different types of cancers, but it is still a largely underexplored facet of MPM. Through the analysis of TCGA data on 86 MPM patients, Pentimalli et al. identify 107 genera signatures that are significantly associated with patient’s survival, thereby suggesting intratumor microbiota both as a novel potential prognostic indicator for MPM and an actionable target for the development of new strategies to improve the efficacy of immunotherapy. Up to date, clinical trials conducted with MPM patients have provided promising results with the combination of ICBs with stereotactic body radiation and chemo-therapy. Along this line, the study of Chang et al. aims to determine the ideal dosing and scheduling of combined treatment with radiotherapy and ICBs. They observe that irradiation of MPM cell lines modulates the expression of immune markers and cytokines that are important for antitumor responses. Consequently, *in vivo* studies should be pursued to gather the mechanisms underlying the synergy between radiotherapy and ICBs. In this regard, it is crucial that preclinical models of mesothelioma improve the accuracy in predicting the response of the human counterpart. For this purpose, Stern et al. characterize the immunobiology of a biphasic mesothelioma model based on intra-peritoneal growth of AB12 cells in immunocompetent mice. Immunologic, transcriptomic, and survival analyses show that intermediate- and advanced-tumors match with human immune active and immunosuppressed MPM, respectively. Therefore, new therapeutics - such as the anti-CTLA-4 + anti-PD-1 + cisplatin triple therapy - showing efficacy at advanced phases, when anti-tumor immune response has decayed, are the most promising candidates to improve outcome in MPM patients.

Additionally, it is important that the research of novel targets and approaches be carried on, in order to increase the number and the efficacy of the therapeutic options for MPM. In this perspective, Digifico et al. find that the protein osteopontin (OPN) is more expressed in human MPM tumors than in normal pleural tissues and is a key promoter of tumor cell proliferation. Accordingly, either the silencing of *OPN* gene in MPM cells or the blocking of its major receptor CD44 by a specific antibody significantly reduce tumor growth *in vivo* in an orthotopic model of MPM. Intriguingly, Chintala et al. performed a phase I clinical trial to evaluate intrapleural administration of oncolytic vaccinia virus, a promising approach for the treatment of solid tumors, in a small cohort of patients with MPM and metastatic disease. Besides being safe and feasible, the study highlights that the genetically engineered vaccinia virus can infect tumor cells and generate immune responses, leading to a decrease in tumor cell density. These results foster further investigation of immunomodulatory effects of oncolytic virus treatment, and their potential clinical implications for combination therapy with ICBs, chemo- or radio- therapy.

Finally, we would like to thank all the authors who have contributed to this Research Topic and the reviewers for their outstanding efforts. We hope that the insights discussed in this Research Topic are not only inspirational for those who are already working in the fields of mesothelioma and immuno oncology, but also captivating and useful for those who are not deeply involved in this Research Topic.

## Author contributions

All authors listed have made a substantial, direct and intellectual contribution to the work, and approved it for publication.

